# Golden Years, Golden Bonds: Exploring the Impact of Retirement on Spouses’ Social Networks and Shared Relationships

**DOI:** 10.1093/geroni/igaf122.839

**Published:** 2025-12-31

**Authors:** Elizabeth Avent, James Ivenuik

**Affiliations:** National Opinion Research Center at The University of Chicago, Chicago, Illinois, United States; National Opinion Research Center at The University of Chicago, Chicago, Illinois, United States

## Abstract

Research has shown that retirement influences spouses’ social networks, though the extent of post-retirement network structure and overlap may differ by gender and ethnicity. Using data from the National Social Life, Health, and Aging Project (NSHAP) (N = 1870) this study examines how spousal retirement affects social networks and shared relationships. Results from generalized structural equation models show that retired men’s networks neither expanded nor diminished but became more interconnected (β = 0.05, p< .05), even as their overall size declined. Additionally, retired men spent less time with their partners (β = -0.42, p<.05), while their retired partners exhibited a similar decrease (β = -0.37, p<.05). Retired women’s networks initially increased (β = 0.15, p< .05) before decreasing over time (β = -0.20, p< .05), with reduced time spent with their network (β = -10.60, p<.05). Although retirement did not significantly alter overall network overlap, greater overlap was observed among Black (β = 32.21, p<.01) and Hispanic (β = 38.63, p<.01) couples when the male retired. Hispanic couples in which the female retired had even higher levels of network overlap (β = 66.26, p<.01). These findings suggest gender and racial/ethnic differences in how couples navigate social networks post-retirement. Understanding these dynamics can inform strategies to promote social engagement and support in later life.

